# Clinical Considerations on HPV Knowledge, Screening, and Vaccination in Brazilian Primary Care: Letter to the Editor

**DOI:** 10.1002/hsr2.73313

**Published:** 2026-09-28

**Authors:** Yudai Kaneda

**Affiliations:** ^1^ Clinical Training Center Jyoban Hospital of Tokiwa Foundation Iwaki Fukushima Japan; ^2^ Health in Humanitarian Crises London School of Hygiene & Tropical Medicine London UK

I read with interest the study by Ramos et al., which identified marked gaps between preventive behavior and understanding among 302 Brazilian women: 69.9% had undergone a Pap smear, but only 19.9% were classified as screened according to the recommended protocol; 72.7% would “certainly” vaccinate their children, yet only 18.5% recognized the association between HPV and cervical cancer [1]. The study provides valuable evidence of favorable HPV vaccination attitudes, but several points may refine the clinical interpretation.

First, the denominator used for screening adherence requires clarification. During the study period, Brazilian guidance recommended cytology for sexually active women aged 25–64 years, initially annually and then every 3 years after two consecutive annual negative tests [2]. However, the reported 19.9% appears to use all 302 participants as the denominator, potentially classifying ineligible women as non‐adherent. Moreover, four women aged ≤ 22 years were categorized as following the protocol, despite being below the routine screening age. I encourage the authors to report adherence among eligible women and distinguish appropriate non‐screening in younger women from under‐screening in the target population.

Second, “certainty about vaccinating children” was reported across the study sample, but the article does not indicate how many participants were mothers or guardians of children within the vaccine‐eligible age range. This matters because 29.2% of women aged ≤ 22 years answered “I do not know yet.” Such responses may reflect hypothetical decision‐making among women without children rather than vaccine hesitancy. Longitudinal evidence indicates that both parental and adolescent intentions may contribute to subsequent uptake [3]. Clarification of parental status and children's ages would improve interpretation; if unavailable, this should be acknowledged as a limitation.

Third, the apparently poorer FHS/PHC evaluations among younger women seem driven by “I do not know/do not remember” responses rather than negative experiences. No woman aged ≤ 22 years selected “Certainly no” when asked whether professionals understood their questions or provided understandable answers, whereas 27.1% and 29.2%, respectively, selected the uncertain response. This pattern may indicate limited primary care exposure rather than lower trust or poorer perceived quality. It may also help explain why healthcare professionals as an information source were not associated with vaccination certainty. Merely receiving information should be distinguished from receiving a clear recommendation, as physician recommendation was the strongest correlate of parental uptake in a meta‐analysis of 62 studies [4]. Likewise, the association with television should not be attributed to the medium itself: Japan's experience illustrates that adverse media framing can undermine HPV vaccine confidence [5].

Finally, several numerical discrepancies merit correction: the abstract reports 96.4% awareness of the Pap smear, whereas Table 3 reports 74.8%; the vaccination‐intention categories total 300 participants despite the stated sample of 302 and the report of no missing data; responses for FHS/PHC as first choice for a new problem total 325 rather than 302; and 39 participants are labeled as 6.0%, although 39/302 is 12.9%.

Addressing these points would strengthen this valuable study and sharpen its clinical and primary care implications.

## Author Contributions

**Yudai Kaneda:** conceptualization, writing – original draft.

## Funding

The author has nothing to report.

## Disclosure

Yudai Kaneda affirms that this manuscript is an honest, accurate, and transparent account of the research reported, that no important aspects of the research have been omitted, and that any differences from the planned research (and registration, if relevant) are explained by Yudai Kaneda.

## Conflicts of Interest

The author declares no conflicts of interest.

## Policy on Using ChatGPT and Similar AI Tools

The author used ChatGPT (OpenAI, San Francisco, CA, USA) to assist with English language editing and translation of the manuscript. The tool was used solely for linguistic refinement. The author takes full responsibility for the content of the manuscript.

## Data Availability

Data sharing is not applicable to this article, as no new data were created or analyzed in this study.

## References

[1] A. M. Ramos , C. V. Bolignani , L. B. D. Göttems , et al., “Knowledge and Awareness of HPV, the HPV Vaccine, and Cervical Cancer Among Women From a Brazilian Community: A Community‐Based Cross‐Sectional Study,” Health Science Reports 9, no. 7 (2026): e72818. 10.1002/hsr2.72818 PMC13369574 42460247

[2] J. C. Teixeira , D. B. Vale , C. S. Campos , J. F. Bragança , M. G. Discacciati , and L. C. Zeferino , “Organization of Cervical Cancer Screening With DNA–HPV Testing Impact on Early–Stage Cancer Detection: A Population–Based Demonstration Study in a Brazilian City,” Lancet Regional Health ‐ Americas 5 (2022): 100084. 10.1016/j.lana.2021.100084 PMC9903591 36776450

[3] V. W. Jongen , M. F. S. van der Loeff , A. Boyd , et al., “Human Papillomavirus Vaccination Uptake: A Longitudinal Study Showing Ethnic Differences in the Influence of the Intention‐to‐Vaccinate Among Parent‐Daughter Dyads,” Human Vaccines & Immunotherapeutics 17, no. 4 (2021): 990–999. 10.1080/21645515.2020.1808411 PMC8018452 32966170

[4] P. A. Newman , C. H. Logie , A. Lacombe‐Duncan , et al., “Parents' Uptake of Human Papillomavirus Vaccines for Their Children: A Systematic Review and Meta‐Analysis of Observational Studies,” BMJ Open 8, no. 4 (2018): e019206. 10.1136/bmjopen-2017-019206 PMC5914890 29678965

[5] Y. Kaneda and M. Namba , “Potential Risk of Overlooking Biased Reporting of Vaccination Against Novel Coronavirus Disease 2019: Lessons From Japan's Experience With the Human Papillomavirus Vaccine,” JMA Journal 6, no. 4 (2023): 513–514. 10.31662/jmaj.2023-0037 PMC10628147 37941685

